# How long should we continue gastric cancer screening? From an epidemiological point of view

**DOI:** 10.1007/s10120-018-0877-z

**Published:** 2018-09-21

**Authors:** Yuri Mizota, Seiichiro Yamamoto

**Affiliations:** Division of Health Sociology, Center for Public Health Sciences, National Cancer Center, 5-1-1 Tsukiji, Chuo, Tokyo, 104-0045 Japan

**Keywords:** Gastric cancer, Cancer screening, Guidelines

## Abstract

**Background:**

In Japan, incidence of gastric cancer is expected to follow the current downward trend as the younger generation has lower incidence of *Helicobacter pylori* infection. In this study we aimed to estimate how long gastric cancer screening is deemed necessary in the future from epidemiologic perspectives.

**Methods:**

Following the Japanese guidelines for gastric cancer screening 2014, recommendation of providing population-based gastric cancer screening is judged by balancing benefits and harms. Benefits and harms are estimated by number needed to screen (NNS) < 1000 and Number Needed to Recall (NNR) < 100. NNS is the number of people required to participate in a screening to prevent one death and NNR is the number of people required to undergo diagnostic examination to prevent one death. These index are estimated for 2020–2035 using future projections of gastric cancer mortality for the scenarios of relative risk (RR) of 0.5–0.9 for mortality reduction by the screening.

**Results:**

The criteria of both NNS < 1000 and NNR < 100 are fulfilled for the following age groups: when RR is set as 0.6, men ≥ 55 and women ≥ 65; when RR is set as 0.7 and 0.8, men ≥ 65 and women ≥ 75; when RR is set as 0.9, men ≥ 75 only.

**Conclusions:**

In case of RR of 0.5 and 0.6, the gastric cancer screening are recommended for men ≥ 55 and women ≥ 65 until 2035, while it is not recommended for men and women in the 45–54 even in 2010 and 2015.

## Introduction

In Japan, incidence of gastric cancer is expected to follow the current downward trend as the younger generation has lower incidence of *Helicobacter pylori* infection [1]. In this study, therefore, we aimed to estimate how long gastric cancer screening is deemed necessary in the future from epidemiologic and statistical perspectives. Of note, for clarification purposes, population-based screening was selected as a screening mode to be analyzed in this study.

In Japan, based on the “Japanese guidelines for gastric cancer screening 2014 edition” edited by the National Cancer Center [2], the Ministry of Health, Labor, and Welfare recommends radiographic screening and endoscopy as population-based screening [3]. Especially, endoscopy screening was recommended very recently since 2016. In principle, population-based screening should be introduced and conducted after comparing and weighing the benefits regarding mortality reduction and harms concerning screening [4, 5]. Even though there are many disagreements over whether performing screenings falling short of such standard is justifiable, few may take a critical attitude toward conducting screenings if they meet this standard. The challenge here is how to compare the benefits, i.e., size of mortality reduction, to the potential harms of screening. The most common harms associated with screening include false-negative test results, false-positive test results, overdiagnosis, as well as adverse reactions to screening and diagnostic examination procedures. It is not easy to compare these issues with the size of mortality reduction effect because they have fundamentally different natures. In the Japanese guidelines for cancer screening 2014 edition, for comparison between benefits and harms of screening, Number Needed to Screen (NNS), representing the size of mortality reduction effect, is used as a benefit indicator, while recall rate is employed as a risk indicator, which is the same as the Japanese guidelines for breast cancer screening [6]. NNS is an estimated number of people required to participate in a screening program to prevent one death over a defined time interval, and thus the smaller NNS implies larger benefits. On the other hand, recall rate is the number of people required to undergo diagnostic examination procedures to prevent one death over a defined time interval, referred as number needed to recall (NNR) in this article, and the larger NNR implies larger harms, i.e., causing inconvenience to more people. In the above-mentioned Guidelines, the thresholds of 1000 and 100 are set as tentative criteria for NNS and NNR, respectively. To judge the length to continue gastric cancer screening, these criteria were used in the present study due to the following facts: these numbers have been employed in the Guidelines in widespread use; using them can allow qualitative analyses; and there are no alternative proven criteria available. In short, we calculate NNS and NNR, compare them to their corresponding threshold of 1000 and 100, and use the comparison results as a part of a basis for deciding whether it is justifiable to continue or discontinue the gastric cancer screening programs.

To maximize the effect of population-based screening, higher participation rate is necessary. Nevertheless, participation rate is as low as 40% in Japan [7] and the government set the goal as 50% in the Third term Basic Plan to Promote Cancer Control Programs in Japan [8]. Since the number of life saved (NLS) varies according to the participation rate, NLS of participation rate 50% and 100% compared to that of NLS of present rate (40%) are also used as a benefit indicator in this study.

## Methods

NNS, NNR, and NLS are estimated by sex and age group. Estimations of NNS, NNR, and NLS require data on gastric cancer mortality, screening effect on mortality reduction, and recall rate. The projections of future gastric cancer deaths by sex and age group in Japan are available from the National Cancer Center [9]. While people are divided into the 7 age groups as follows: 0–14, 15–44, 45–54, 55–64, 65–74, older than or equal to 75 years of age, and all ages, we selected age groups at the time of screening as follows: 45–54, 55–64, 65–74, and older than or equal to 75 years of age in our study. In addition to the number of deaths, estimations of mortality rates require estimates of future population, which should be calculated using the same method and numbers used for calculation of the number of deaths, and thus, we used the method described in the reference [10]. However, since there is no publicly disclosed prediction for the future Japanese population in the period of 2015 and beyond, a ratio of Japanese population to the total population in Japan by sex and 5-year age groups were calculated, which in turn was multiplied by the total population estimates (estimated median numbers of births and deaths) for the year of 2020, 2025, 2030, and 2035, to obtain estimates of future Japanese population by sex and 5-year age groups. These data on the Japanese total population are published by The National Institute of Population and Social Security Research [11]. The projections of the gastric cancer mortality rates are estimated for 2020, 2025, 2030, and 2035 using future number of deaths estimates of 2020–2024, 2025–2029, 2030–2034, and 2035–2039, respectively. Mortality trends are shown using observed value until 2015 [12] and estimates for 2020–2035.

To estimate NNS, the above-mentioned Guidelines used relative risks (RR) of gastric cancer mortality reduction for effectiveness of radiography test and endoscopy test from several studies [13–15]. In this study, several relative risk values associated with screening are used for estimation of future NNSs and NNRs in different scenarios. For reference, Table 1 lists the relative risk values used in the Guidelines. These relative risk values ranged from 0.1 to 1.07, which included those either too large or too small to exert any effects, and thus 5 values (0.5, 0.6, 0.7, 0.8, and 0.9) were selected to be used in the scenarios in this study. Recently Korean study reported that the effectiveness of endoscopy screening is RR of 0.53 (95% CI 0.51–0.56), which is not contradict from our scenarios [16].

**Table 1:** Relative risk used to estimate number needed to screen in the Japanese guidelines for gastric cancer

Screening	Study	Sex	Age-specific relative risk							
			40	45	50	55	60	65	70	75
Radiography	Abe et al. [13]	Male	0.105	0.105	0.25	0.25	0.271	0.271	0.429	0.429
		Female	0.778	0.778	0.2	0.2	0.385	0.385	0.882	0.882
	Fukao et al. [14]	Male			0.46	0.46	0.34	0.34	0.25	0.25
		Female			1.07	1.07	0.45	0.45	0.63	0.63
	Hamashima et al. [15]	Male	0.865	0.865	0.865	0.865	0.865	0.865	0.865	0.865
		Female	0.865	0.865	0.865	0.865	0.865	0.865	0.865	0.865
Endoscopy	Hamashima et al. [15]	Male	0.695	0.695	0.695	0.695	0.695	0.695	0.695	0.695
		Female	0.695	0.695	0.695	0.695	0.695	0.695	0.695	0.695

Japanese Guidelines for Gastric Cancer 2014 edition. http://canscreen.ncc.go.jp/

Recall rates cited in the above-mentioned Guidelines are radiography test data derived from the annual report 2011 of The Japanese Society of Gastrointestinal Cancer Screening [17], and endoscopy data collected in Niigata City reported in 2012 [18] (Table 2). The ranges of recall rates for radiography test and endoscopy were reported as 4.1–12.2% and 2.9–11.6%, respectively. In this study, we used relative risks of 5% and 10% as scenarios.

**Table 2:** Recall rate used to estimate number needed to recall in the Japanese guidelines for gastric cancer

Screening	Study	Sex	Age-specific recall rate (%)							
			40	45	50	55	60	65	70	75
Radiography	JSGCS [17]	Male	4.8	6.0	7.9	9.8	11.3	11.9	12.2	12.2
		Female	4.1	4.72	5.7	6.5	7.3	7.9	8.5	8.5
Endoscopy	Niigata City [18]	Male	2.9	8.9	11.6	9.7	11.5	11.0	11.2	11.2
		Female	5.8	5.4	6.4	6.7	7.5	7.3	7.3	7.3

Japanese Guidelines for Gastric Cancer 2014 edition. http://canscreen.ncc.go.jp/

For estimating NLS, hypothetical number of gastric cancer deaths without screening, *D*_0s_, is estimated as follows:

$${\hat {D}_0}=\frac{{{D_{{\text{obs}}}}}}{{1 - {P_{{\text{obs}}}}\left( {1 - {\text{RR}}} \right)}},$$

where *D*_obs_ is observed number of deaths and *P*_obs_ is observed participation rate of screening. NLS_t_ is estimated as a function of target participation rate *P*_t_:

$$N\hat {L}{S_t}={D_0}\left( {1 - {P_t}\left( {1 - {\text{RR}}} \right)} \right).$$

The observed participation rate is set as 40% and target participation rates are set as 50% and 100%. For the future predication, *P*_obs_ is assumed as the same as the present participation rate, i.e., 40%.

## Results

**Fig. 1:**
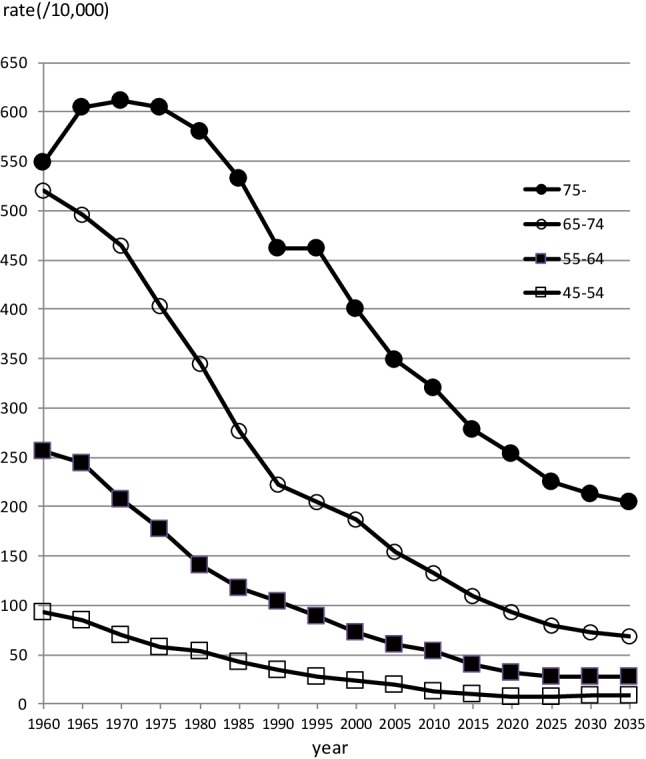
Observed and projected trends of age-specific gastric cancer mortality in Japan for male

**Fig. 2:**
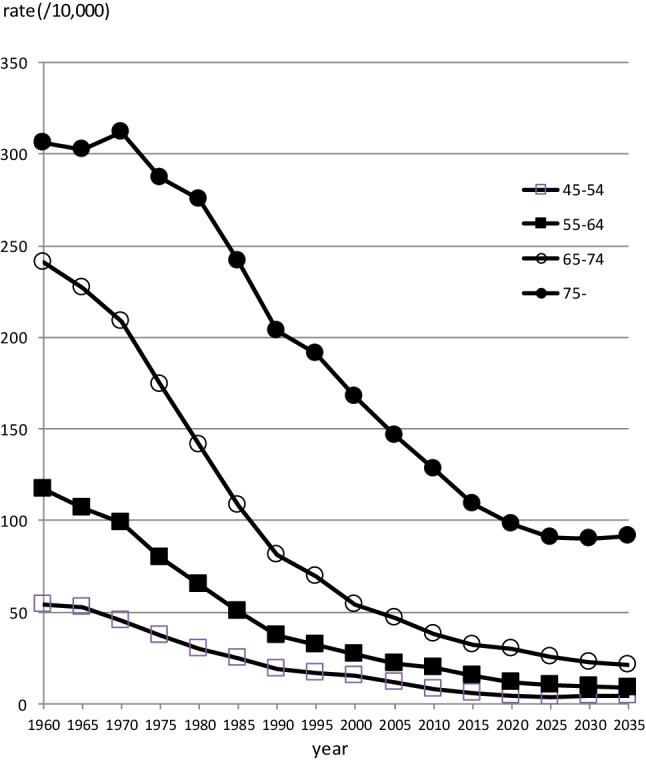
Observed and projected trends of age-specific gastric cancer mortality in Japan for female

Figures 1 and 2 show past transition and future projections of gastric cancer mortalities by age groups. Downward trends are obvious for both men and women in every age group equal to and older than 45 years old.

Tables 3 and 4 show estimates of NNS and NNR. It might be obvious, but higher relative risks (small effect) and/or lower mortality rates make NNS higher. The results indicated that the benefits of the screening exceeded harms more prominently in men than women, older than younger age groups, and now than future. The criteria of both NNS and NNR would be fulfilled, that is, the both benefits and harms are considered within acceptable limits to justify the screening, for the following age groups (year-old): when relative risk (RR) of screening is set as 0.5, men ≥ 55 and women ≥ 65; when RR is set as 0.6, men ≥ 55 and women ≥ 65; when RR is set as 0.7, men ≥ 65 and women ≥ 75; and when RR is set as 0.8, men ≥ 65 and women ≥ 75; when RR is set as 0.9, men ≥ 75 only.

**Table 3:** Number needed to screen, number needed to recall, and number of life saved by gastric cancer screening based on future prediction of gastric cancer mortality

Mortality reduction^a^	Year	Age 45–54						Age 55–64						Age 65–74						Age 75-					
		Mortality rate for 10 years^b^ (%)	NNS^c^	NNR^d^		NLS^5^		Mortality rate for 10 years (%)	NNS	NNR		NLS		Mortality rate for 10 years (%)	NNS	NNR		NLS		Mortality rate for 10 years (%)	NNS	NNR		NLS	
				Recall rate		Participation rate				Recall rate		Participation rate				Recall rate		Participation rate				Recall rate		Participation rate	
				5%	10%	50%	100%			5%	10%	50%	100%			5%	10%	50%	100%			5%	10%	50%	100%
RR^d^ = 0.5	2010	0.13	1560	78	156	63	376	0.54	372	19	37	310	1861	1.32	151	8	15	593	3559	3.21	62	3	6	1071	6428
	2015	0.09	2140	107	214	49	293	0.40	497	25	50	199	1197	1.09	184	9	18	565	3393	2.79	72	4	7	1095	6572
	2020	0.07	2709	135	271	43	255	0.31	643	32	64	147	881	0.93	214	11	21	486	2914	2.54	79	4	8	1165	6990
	2025	0.10	2725	136	273	41	248	0.33	706	35	71	143	855	0.89	252	13	25	354	2126	2.55	89	4	9	1229	7376
	2030	0.08	2503	125	250	39	233	0.28	725	36	73	153	919	0.72	278	14	28	308	1849	2.13	94	5	9	1224	7346
	2035	0.09	2210	110	221	39	233	0.27	732	37	73	148	889	0.69	290	14	29	317	1901	2.04	98	5	10	1148	6885
RR = 0.6	2010	0.13	1950	97	195	48	287	0.54	465	23	47	236	1418	1.32	189	9	19	452	2711	3.21	78	4	8	816	4897
	2015	0.09	2675	134	268	37	223	0.40	621	31	62	152	912	1.09	230	11	23	431	2585	2.79	90	4	9	835	5007
	2020	0.07	3386	169	339	32	194	0.31	804	40	80	112	671	0.93	268	13	27	370	2220	2.54	98	5	10	888	5326
	2025	0.10	3407	170	341	31	189	0.33	882	44	88	109	651	0.89	315	16	32	270	1620	2.55	111	6	11	937	5620
	2030	0.08	3129	156	313	30	177	0.28	906	45	91	117	700	0.72	348	17	35	235	1409	2.13	117	6	12	933	5597
	2035	0.09	2762	138	276	30	177	0.27	915	46	92	113	677	0.69	362	18	36	241	1449	2.04	123	6	12	874	5246
RR = 0.7	2010	0.13	2600	130	260	34	205	0.54	620	31	62	169	1015	1.32	252	13	25	324	1941	3.21	104	5	10	584	3506
	2015	0.09	3567	178	357	27	160	0.40	829	41	83	109	653	1.09	306	15	31	308	1851	2.79	120	6	12	597	3585
	2020	0.07	4515	226	452	23	139	0.31	1072	54	107	80	481	0.93	357	18	36	265	1589	2.54	131	7	13	635	3813
	2025	0.10	4542	227	454	23	135	0.33	1176	59	118	78	466	0.89	420	21	42	193	1160	2.55	148	7	15	671	4023
	2030	0.08	4171	209	417	21	127	0.28	1208	60	121	84	501	0.72	463	23	46	168	1008	2.13	156	8	16	668	4007
	2035	0.09	3683	184	368	21	127	0.27	1220	61	122	81	485	0.69	483	24	48	173	1037	2.04	163	8	16	626	3755
RR = 0.8	2010	0.13	3900	195	390	22	131	0.54	930	47	93	108	647	1.32	378	19	38	206	1238	3.21	156	8	16	373	2236
	2015	0.09	5351	268	535	17	102	0.40	1243	62	124	69	416	1.09	459	23	46	197	1180	2.79	179	9	18	381	2286
	2020	0.07	6773	339	677	15	89	0.31	1608	80	161	51	307	0.93	535	27	54	169	1013	2.54	197	10	20	405	2431
	2025	0.10	6814	341	681	14	86	0.33	1765	88	176	50	297	0.89	630	32	63	123	740	2.55	222	11	22	428	2566
	2030	0.08	6257	313	626	13	81	0.28	1813	91	181	53	320	0.72	695	35	70	107	643	2.13	234	12	23	426	2555
	2035	0.09	5525	276	552	13	81	0.27	1830	92	183	52	309	0.69	724	36	72	110	661	2.04	245	12	25	399	2395
RR = 0.9	2010	0.13	7799	390	780	10	63	0.54	1860	93	186	52	310	1.32	755	38	76	99	593	3.21	312	16	31	179	1071
	2015	0.09	10702	535	1070	8	49	0.40	2486	124	249	33	199	1.09	918	46	92	94	565	2.79	359	18	36	183	1095
	2020	0.07	13546	677	1355	7	43	0.31	3215	161	322	24	147	0.93	1071	54	107	81	486	2.54	394	20	39	194	1165
	2025	0.10	13627	681	1363	7	41	0.33	3529	176	353	24	143	0.89	1261	63	126	59	354	2.55	444	22	44	205	1229
	2030	0.08	12514	626	1251	6	39	0.28	3625	181	363	26	153	0.72	1390	70	139	51	308	2.13	469	23	47	204	1224
	2035	0.09	11050	552	1105	6	39	0.27	3661	183	366	25	148	0.69	1448	72	145	53	317	2.04	490	25	49	191	1148

^a^Relative risk for mortality reduction by screening

^b^Gastric cancer mortality rate fro 10 years

^c^Number needed to screen

^d^Number needed to recall

^e^Number of life saved

**Table 4:** Number needed to screen, number needed to recall, and number of life saved by gastric cancer screening based on future prediction of gastric cancer mortality

Mortality reduction^a^	Year	Age 45–54						Age 55–64						Age 65–74						Age 75-					
		Mortality rate for 10 years^b^ (%)	NNS^c^	NNR^d^		NLS^e^		Mortality rate for 10 years (%)	NNS	NNR		NLS		Mortality rate for 10 years (%)	NNS	NNR		NLS		Mortality rate for 10 years (%)	NNS	NNR		NLS	
				Recall rate		Participation rate				Recall rate		Participation rate				Recall rate		Participation rate				Recall rate		Participation rate	
				5%	10%	50%	100%			5%	10%	50%	100%			5%	10%	50%	100%			5%	10%	50%	100%
RR = 0.5	2010	0.08	2497	125	250	39	233	0.20	1019	51	102	116	698	0.38	525	26	53	192	1149	1.28	156	8	16	706	4236
	2015	0.06	3339	167	334	31	184	0.15	1304	65	130	77	465	0.32	617	31	62	186	1113	1.09	183	9	18	680	4081
	2020	0.04	4893	245	489	23	139	0.12	1722	86	172	56	334	0.30	675	34	68	168	1009	0.98	204	10	20	694	4166
	2025	0.04	5198	260	520	21	128	0.10	1980	99	198	51	308	0.25	792	40	79	123	735	0.91	221	11	22	736	4418
	2030	0.04	5090	255	509	19	113	0.09	2124	106	212	53	315	0.23	887	44	89	104	623	0.90	222	11	22	768	4609
	2035	0.04	4791	240	479	18	105	0.09	2234	112	223	49	293	0.21	959	48	96	102	611	0.92	218	11	22	775	4650
RR = 0.6	2010	0.08	3121	156	312	30	177	0.20	1273	64	127	89	532	0.38	656	33	66	146	876	1.28	195	10	19	538	3227
	2015	0.06	4174	209	417	23	140	0.15	1630	82	163	59	354	0.32	771	39	77	141	848	1.09	229	11	23	518	3109
	2020	0.04	6117	306	612	18	106	0.12	2153	108	215	42	254	0.30	844	42	84	128	769	0.98	255	13	25	529	3174
	2025	0.04	6497	325	650	16	97	0.10	2475	124	247	39	234	0.25	990	50	99	93	560	0.91	276	14	28	561	3366
	2030	0.04	6363	318	636	14	86	0.09	2655	133	266	40	240	0.23	1109	55	111	79	474	0.90	277	14	28	585	3511
	2035	0.04	5989	299	599	13	80	0.09	2792	140	279	37	223	0.21	1199	60	120	78	466	0.92	273	14	27	590	3543
RR = 0.7	2010	0.08	4161	208	416	21	127	0.20	1698	85	170	63	381	0.38	875	44	88	104	627	1.28	260	13	26	385	2311
	2015	0.06	5565	278	557	17	100	0.15	2173	109	217	42	253	0.32	1028	51	103	101	607	1.09	306	15	31	371	2226
	2020	0.04	8156	408	816	13	76	0.12	2870	144	287	30	182	0.30	1125	56	113	92	550	0.98	339	17	34	379	2273
	2025	0.04	8663	433	866	12	70	0.10	3300	165	330	28	168	0.25	1320	59	132	67	401	0.91	368	18	37	402	2410
	2030	0.04	8484	424	848	10	61	0.09	3541	177	354	29	172	0.23	1478	74	148	57	340	0.90	370	18	37	419	2514
	2035	0.04	7985	399	799	10	57	0.09	3723	186	372	27	160	0.21	1599	80	160	56	333	0.92	363	18	36	423	2536
RR = 0.8	2010	0.08	6241	312	624	14	81	0.20	2546	127	255	40	243	0.38	1313	66	131	67	400	1.28	390	19	39	246	1473
	2015	0.06	8348	417	835	11	64	0.15	3260	163	326	27	162	0.32	1542	77	154	65	387	1.09	459	23	46	237	1420
	2020	0.04	12234	612	1223	8	48	0.12	4306	215	431	19	116	0.30	1688	84	169	58	351	0.98	509	25	51	242	1449
	2025	0.04	12994	650	1299	7	44	0.10	4949	247	495	18	107	0.25	1980	99	198	43	256	0.91	551	28	55	256	1537
	2030	0.04	12726	636	1273	7	39	0.09	5311	266	531	18	110	0.23	2218	111	222	36	217	0.90	554	28	55	267	1603
	2035	0.04	11978	599	1198	6	37	0.09	5584	279	558	17	102	0.21	2398	120	240	35	213	0.92	545	27	55	270	1617
RR = 0.9	2010	0.08	12483	624	1248	6	39	0.20	5093	255	509	19	116	0.38	2626	131	263	32	192	1.28	779	39	78	118	706
	2015	0.06	16695	835	1670	5	31	0.15	6520	326	652	13	77	0.32	3083	154	308	31	186	1.09	917	46	92	113	680
	2020	0.04	24467	1223	2447	4	23	0.12	8611	431	861	9	56	0.30	3376	169	338	28	168	0.98	1018	51	102	116	694
	2025	0.04	25988	1299	2599	4	21	0.10	9899	495	990	9	51	0.25	3960	198	396	20	123	0.91	1103	55	110	123	736
	2030	0.04	25452	1273	2545	3	19	0.09	10622	531	1062	9	53	0.23	4435	222	444	17	104	0.90	1109	55	111	128	768
	2035	0.04	23955	1198	2396	3	18	0.09	11169	558	1117	8	49	0.21	4796	240	480	17	102	0.92	1090	55	109	129	775

^a^Relative risk for mortality reduction by screening

^b^Gastric cancer mortality rate fro 10 years

^c^Number needed to screen

^d^Number needed to recall

^e^Number of life saved

NLS, which is a function of RR, mortality, and participation rate, is substantial for age 65 or older when participation rate is 50% as a national goal while it is not so large for either two combination of female, RR ≥ 0.8, and age 54 or younger.

## Discussion

In this study, target population and length appropriate to continue gastric cancer screening were investigated based on the future projection of gastric cancer mortality, from the standpoint of balancing the benefits and harms of the screening. As a result, until 2035, screening programs with higher mortality reduction effects (relative risk 0.5 and 0.6) are shown to be beneficial for men ≥ age 55 and women ≥ age 65. It is expected that, under conditions and scenarios selected in this study, both men and women in the 45–54 age group did not meet the criteria for benefits and harms even in 2010 and 2015.

This study can provide evidence for the decision based on benefits and harms by numerical criteria using NNS, NNR, and NSL. In this way, balancing estimates of benefits and harms is a standard method to evaluate whether to introduce and continue population-based screening [5, 19, 20]. While more comprehensive balance sheets have been proposed [21, 22], typical indicators are those for concerning mortality reduction for benefit and false-positive, overdiagnosis, and adverse reactions to screening and diagnostic examination procedures for harm [19, 20, 23]. The NNS and NNR used in this study are transformed indictors of mortality reduction and false-positive for intuitive interpretation. Overdiagnosis indicators cannot be examined due to lack of reports about overdiagnosis for gastric cancer screening [2]. Because of the difficulty of comparing severity of adverse reactions with screening benefit in numerical way, NNS and NNR were used to balance benefits and harms in this study. As for the threshold, no consensus was obtained due to the uncertainty and variability in the evidence used to make these estimates [20] or a matter of individual judgement [19]. In this study, we used threshold of 1000 for NNS and 100 for NNR based on the Japanese guidelines for cancer screening 2014 edition [2]. These threshold has some sense in Japan because the recommendation of the guideline and following government decision was made based on this value. Even in case of not using such threshold, combination of NNS and NNR for various scenarios in Tables 3 and 4 will help to evaluate whether to continue gastric cancer screening.

There are several limitations in this study. NNSs, NNRs, and NLS addressed in this study are limited to those estimated using the data obtained for both male and female in the age groups of 45–54, 55–64, 65–74, and equal to and older than 75 years, projected for 2020, 2025, 2030, and 2035, due to limited availability of the relevant data. The accurate data of the effect size of screening on mortality, recall rate, and participation rate are not available in Japan, while the detailed and accurate data on mortality rates and their projections were available. Unfortunately, however, although stomach cancer screening has been recommended for age 40 or older until 2015 and is recommended for age 50 or older since 2016, the projections are only available for age groups of 45–54, 55–64, 65–74, and equal to and older than 75 years old. Although NNSs, NNRs, and NLSs outside of these scenarios cannot be estimated due to data availability, they can be speculated by intrapolation of the values of mortality rate, relative risk, and recall rate within the scenarios. Owing to the simple relationships among these values, the results can be speculated that gastric cancer screening is not recommended for men and women with age 50 based on the threshold of NNS < 1000 and NNR > 100 for all the scenarios (Tables 3, 4). As a matter of course, in real situations, other benefits and harms of the screening should be considered such as less invasive treatment due to early detection as benefits and adverse reactions of the screening and diagnostic examinations as harms.

Considering the criteria of benefits and harms as NNS < 1000 and NNR > 100, respectively, these estimates may imply that, compared to sex, age and screening effect, the trend toward mortality reduction may have less impact on NNS and NNR, at least until 2035. Recall rates are closely related to prevalence, sensitivity, specificity, and screening effect, and therefore, it is important to manage the accuracy level of screening to maintain the recall rates in reasonable range. Furthermore, NLS heavily depends on participation rate of screening, it is most important to increase participation rate as high as possible.
